# The Prevalence of Non-Alcoholic Fatty Liver Disease in Children and Adolescents: A Systematic Review and Meta-Analysis

**DOI:** 10.1371/journal.pone.0140908

**Published:** 2015-10-29

**Authors:** Emma L. Anderson, Laura D. Howe, Hayley E. Jones, Julian P. T. Higgins, Debbie A. Lawlor, Abigail Fraser

**Affiliations:** 1 MRC Integrative Epidemiology Unit at the University of Bristol, Bristol, United Kingdom; 2 School of Social and Community Medicine, University of Bristol, Bristol, United Kingdom; 3 Centre for Reviews and Dissemination, University of York, York, United Kingdom; The Chinese University of Hong Kong, HONG KONG

## Abstract

**Background & Aims:**

Narrative reviews of paediatric NAFLD quote prevalences in the general population that range from 9% to 37%; however, no systematic review of the prevalence of NAFLD in children/adolescents has been conducted. We aimed to estimate prevalence of non-alcoholic fatty liver disease (NAFLD) in young people and to determine whether this varies by BMI category, gender, age, diagnostic method, geographical region and study sample size.

**Methods:**

We conducted a systematic review and meta-analysis of all studies reporting a prevalence of NAFLD based on any diagnostic method in participants 1–19 years old, regardless of whether assessing NAFLD prevalence was the main aim of the study.

**Results:**

The pooled mean prevalence of NAFLD in children from general population studies was 7.6% (95%CI: 5.5% to 10.3%) and 34.2% (95% CI: 27.8% to 41.2%) in studies based on child obesity clinics. In both populations there was marked heterogeneity between studies (I^2^ = 98%). There was evidence that prevalence was generally higher in males compared with females and increased incrementally with greater BMI. There was evidence for differences between regions in clinical population studies, with estimated prevalence being highest in Asia. There was no evidence that prevalence changed over time. Prevalence estimates in studies of children/adolescents attending obesity clinics and in obese children/adolescents from the general population were substantially lower when elevated alanine aminotransferase (ALT) was used to assess NAFLD compared with biopsies, ultrasound scan (USS) or magnetic resonance imaging (MRI).

**Conclusions:**

Our review suggests the prevalence of NAFLD in young people is high, particularly in those who are obese and in males.

## Introduction

Non-alcoholic fatty liver disease (NAFLD) is defined as the accumulation of fat in the liver in the absence of excessive alcohol consumption or other known liver pathologies.[1] It is a spectrum of disease ranging from steatosis (fat infiltration into the liver) to steatohepatitis, which is characterised by hepatocellular inflammation and injury, to fibrosis and eventually cirrhosis.[2,3] NAFLD is now recognised as one of the most common causes of chronic liver disease in young people in the developed world.[4,5]

The prevalence of NAFLD in adults and children in the general population is uncertain and difficult to assess accurately due to a lack of simple, non-invasive diagnostic tests.[6] The ‘gold standard’ for diagnosing NAFLD and its severity is a liver biopsy, but this is neither feasible nor ethical to use in healthy populations. Even in clinical practice, liver biopsies are used for clarity of chronic hepatitis (for example, in patients with indeterminate or discordant results unable to exclude advanced fibrosis).[7] Consequently, population prevalence is usually estimated by serum biomarkers of NAFLD and/or evidence of fatty liver on USS or magnetic resonance imaging (MRI).

The commonest serum biomarkers used to assess NAFLD prevalence in research settings and to identify patients who may benefit from further investigation in clinical settings are alanine aminotransferase (ALT) and aspartate aminotransferase (AST).[8] There is currently no consensus on the thresholds of liver enzymes that should be used to indicate NAFLD or whether thresholds should be specific to sex, age and/or ethnicity.[9–15] Both liver enzymes have been reported to correlate with the degree of liver fat infiltration, inflammation [16–20], in adults and children, though not consistently with fibrosis[5]. However, estimated sensitivity and specificity of these serum indicators for identifying NAFLD remains low compared with histology and imagining techniques.[5] One study of obese children estimated sensitivity and specificity for the prediction of NAFLD with ALT >30U/L to be 64% and 81%, respectively and with ALT >40U/L to be 41% and 89%, respectively compared to a hepatic fat fraction >9% on MRI.[15] USS is the most commonly used imaging modality for determining NAFLD prevalence in the general population as it is relatively inexpensive and, when compared to liver biopsy, has good estimated sensitivity (85%) and specificity (94%) for diagnosing moderate to severe steatosis.[21] It is less reliable for detecting mild steatosis.[22] Compared with liver biopsy, MRI has high estimated sensitivity and specificity for diagnosing steatosis across the whole spectrum of disease (sensitivity and specificity ranges for detecting mild to severe steatosis: 82–97% and 76–95%, respectively). However, MRI is expensive and therefore not often used in general population studies.[22]

Whilst narrative reviews of paediatric NAFLD quote prevalence estimates in the general population that range from 9 to 37%,[2,23] to date, no systematic review of the prevalence of NAFLD in children/adolescents has been conducted. Therefore, the true prevalence of the disease and how this varies over time and with age, gender, geographical region, obesity status and assessment method is unknown. Here we report results of a systematic review and meta-analysis of studies reporting the prevalence of NAFLD in children and adolescents aged between 1 and 19 years, in the general population and in populations of clinically obese adolescents. We aimed to estimate the prevalence of NAFLD in young people and to determine whether and how this varies over time, by body mass index (BMI) category, gender, age, diagnostic method, geographical region and study sample size.

## Methods

### Study eligibility and identification

Studies were eligible for the review if they measured prevalence of NAFLD based on any diagnostic method (i.e. biopsy, USS, MRI or other scans or liver enzymes) in participants aged between 1 and 19 years. Studies using blood-based biomarkers to estimate prevalence were eligible irrespective of the liver enzyme and threshold that was used to define NAFLD. Studies of participants with previous or existing liver disease, for example, if the sample were a group of children that had been selected for further analysis because of hepatomegaly or elevated transaminases, were not eligible because we were unable to obtain a prevalence estimate for them (i.e. because all participants had some evidence of liver disease, we had no denominator with which to estimate prevalence). Diagnosis of NAFLD did not require studies to have excluded alcohol-related fatty liver disease.

A systematic search was conducted in PubMed (US National Library of Medicine, National Institutes of Health) to identify all potentially relevant publications up to October 2013. Our search strategy had two components, the first including terms related to NAFLD and the second including terms that would identify studies specifically in children or adolescents. Search terms entered into PubMed are detailed in S1 File. The search was restricted to humans. Reference lists of all papers identified as relevant were checked for further relevant studies.

### Study and data selection

One author (EA) reviewed all titles retrieved by the search, excluded clearly irrelevant publications and screened all remaining abstracts for eligibility. Full text papers of those potentially eligible were obtained for further evaluation. Papers were discarded if they did not present original data (if two studies published on the same cohort, the largest study was considered). We excluded studies when the publications were inaccessible from any British library or we were unable to obtain translations into English. General population studies were excluded if they did not use random samples (for example, studies where obese cases and lean controls were selected from a general population were excluded) so as not to over- or under-represent a particular BMI category.

### Data extraction

**Fig 1:**
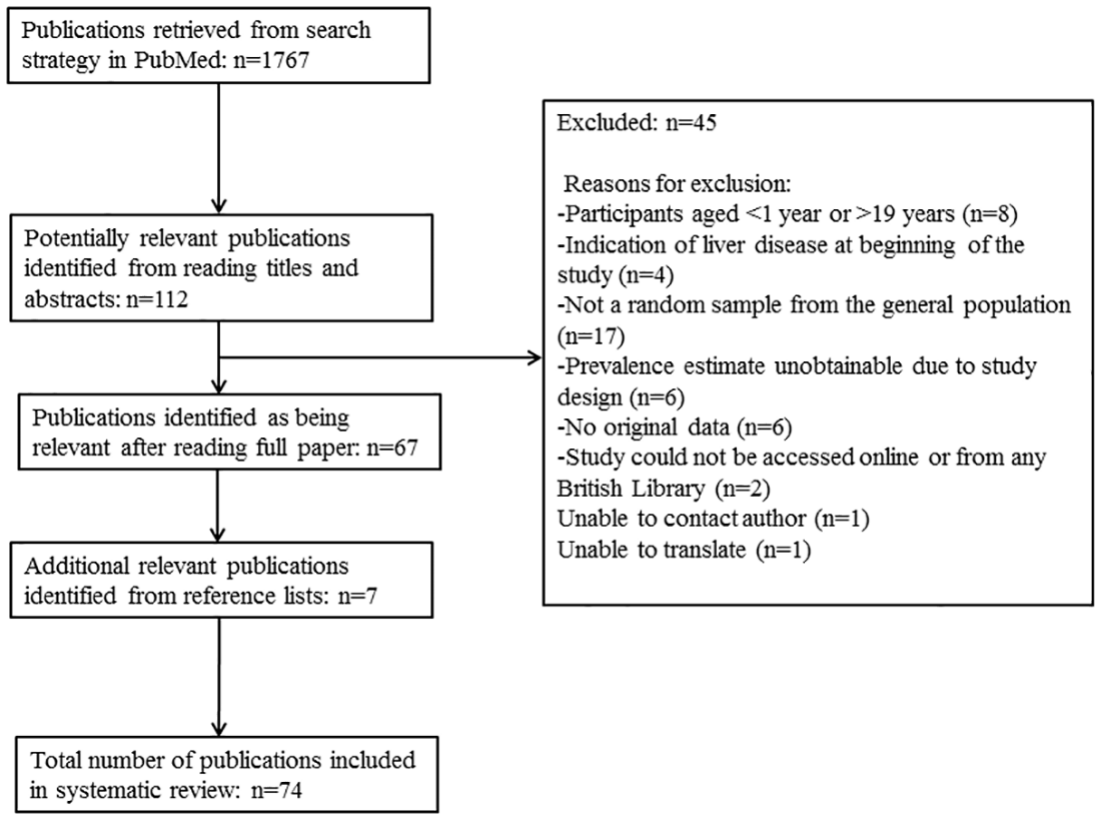
Study Selection.

Following the identification of all potentially relevant papers, data were extracted by EA and one of DAL, LDH or AF, so that two investigators reviewed each paper independently using a standardised extraction form. Any discrepancies in the extractions were discussed and an agreement was made by consensus. Data from non-English language papers were extracted by colleagues who were fluent in the relevant language. One publication in Japanese that could not be translated was excluded from this review (Fig 1). Where possible, the prevalence of NAFLD was extracted for the overall study sample (with males and females combined) and separately by gender. In general population studies we also extracted NAFLD prevalence by BMI category (i.e. normal weight, overweight and obese) where possible. For intervention studies the pre-intervention prevalence was recorded. In some studies the prevalence of NAFLD was not reported directly but could be calculated from published tables and figures. Study authors were contacted for additional data or clarification when required.

### Meta-analytical methods for estimating the prevalence of NAFLD

All analyses were conducted in Stata MP Version 13.1. Meta-analyses were conducted to examine prevalence in the general population and for clinical obese populations of children/adolescents. Several studies of clinical obese populations used more than one diagnostic method to estimate the prevalence of NAFLD (for example, some studies reported two separate NAFLD prevalence estimates by USS and by elevated ALT). We randomly selected one of the methods to include in analyses and conducted a sensitivity analysis to check whether the overall prevalence in clinical obese population studies differed when alternative diagnostic methods were used.

As NAFLD is strongly associated with obesity,[24,25] we decided *a priori* to analyse studies with random samples from the general population separately from those conducted in clinical obese populations (i.e. where participants were recruited through their attendance at a primary or secondary care obesity service). Children seen by a physician because of their obesity are likely to be different from those children (even with the same BMI) in the general population who have not been referred to a clinical obesity team.

The logit transformation was applied to prevalence proportions to better approximate a normal distribution, and pooled logit prevalence estimates were back transformed to their original scale for ease of interpretation. As a sensitivity analysis we also assessed results when an arcsine transformation was used instead of a logit transformation.[26,27] A random-effects model was used to estimate the average prevalence across studies, with 95% confidence limits. Heterogeneity was quantified using estimates of I^2^, which is the percentage of the total observed variability that is due to true prevalence differences between studies rather than chance variation.[28] We also present 95% prediction intervals, which portray the extent of heterogeneity by providing the range in which we would expect the prevalence of NAFLD to lie in a new study, 95% of the time.[29]

### Assessing differences in NAFLD prevalence by BMI, gender, age, publication year, diagnostic method, geographical region and study sample size

A subset of studies reported prevalence estimates stratified by gender or by BMI category. We performed meta-analyses for each of these subgroups separately to provide estimates of prevalence for each gender or BMI category. In addition, to formally assess whether there was evidence of a difference in prevalence by gender or BMI, we compared NAFLD prevalence within studies: (i) in males compared with females and (ii) per increase in BMI category (i.e. assuming a linear association between BMI category and log-odds of NAFLD). We then pooled these within-study estimates (differences in logit prevalences or, equivalently, log odds ratios) using random effects meta-analysis, producing summary odds ratios. Since the assumption of linearity may be questionable, we also obtained the pooled NAFLD odds ratio for obese participants compared with normal and overweight (combined) and for obese and overweight (combined) participants compared with normal weight participants. This allowed us to check that any estimated association was robust to the linearity assumption.

Univariable meta-regression was used to assess whether the following study-level characteristics were associated with NAFLD prevalence in either general or clinical population studies: diagnostic method (categorised as ALT, USS or MRI), average age of participants (categorised as 0 ≤15 and >15 years), publication year (before 2005, 2005 to 2010 and after 2010), geographical region of study (categorised as Europe, Asia, Middle East and North Africa, North America, South America and Oceania), and sample size (categorised as above or below the median sample size: n = 321 for general population studies and n = 77 for clinical studies). Estimates of the average prevalence with 95% CIs in each subgroup were calculated from meta-regression coefficients. P values for differences between subgroups are also reported. In addition to examining the association between publication year and prevalence using univariable meta-regression analyses, we also performed sensitivity analyses in which only those studies published within the last five years were included.

## Results

The search retrieved 1767 publications of which 74 are included in this systematic review, corresponding to 76 independent study populations, as there were two studies which presented prevalence estimates from surveys of two different study samples at different points in time (Fig 1). A full description of included studies is provided in **Tables A** (general population studies) and **B** (clinical population studies) in S1 File. Full references for each study are also provided in S1 File. Sample sizes ranged from 7 to 6,895; median n = 87. The oldest study was published in 1995. The main aim of the majority of studies was to examine associations of NAFLD with potential risk factors and/or outcomes. Overall, 18 studies (20 independent study populations) were conducted in general populations and 56 studies in clinical populations of obese children/adolescents (56 independent study populations). Although the prevalence estimates extracted for this systematic review were reported by studies of various designs (e.g. cross sectional, prospective and intervention studies), prevalence estimates themselves are, by nature, cross-sectional.

The most commonly used diagnostic method in general population studies was USS (n = 10/18, 56% of studies), followed by elevated ALT (n = 8/18, 44% of studies). One study used more than one method and reported prevalence estimates from each. Only one general population study had biopsy data from autopsies and no studies used MRI or computed tomography. In studies of clinical populations of obese children/adolescents, USS was the most common method for assessing NAFLD (n = 41/56, 73%), followed by elevated ALT (n = 19/56, 34%) and MRI (n = 9/56, 16%). Fourteen studies used more than one method and reported prevalence estimates from each. One study of obese clinical populations had biopsy data. The most common ALT threshold used to diagnose NAFLD in both types of study was 40 U/l (5 of 9 general population studies and 7 of 19 clinical studies used this threshold).

**Fig 2:**
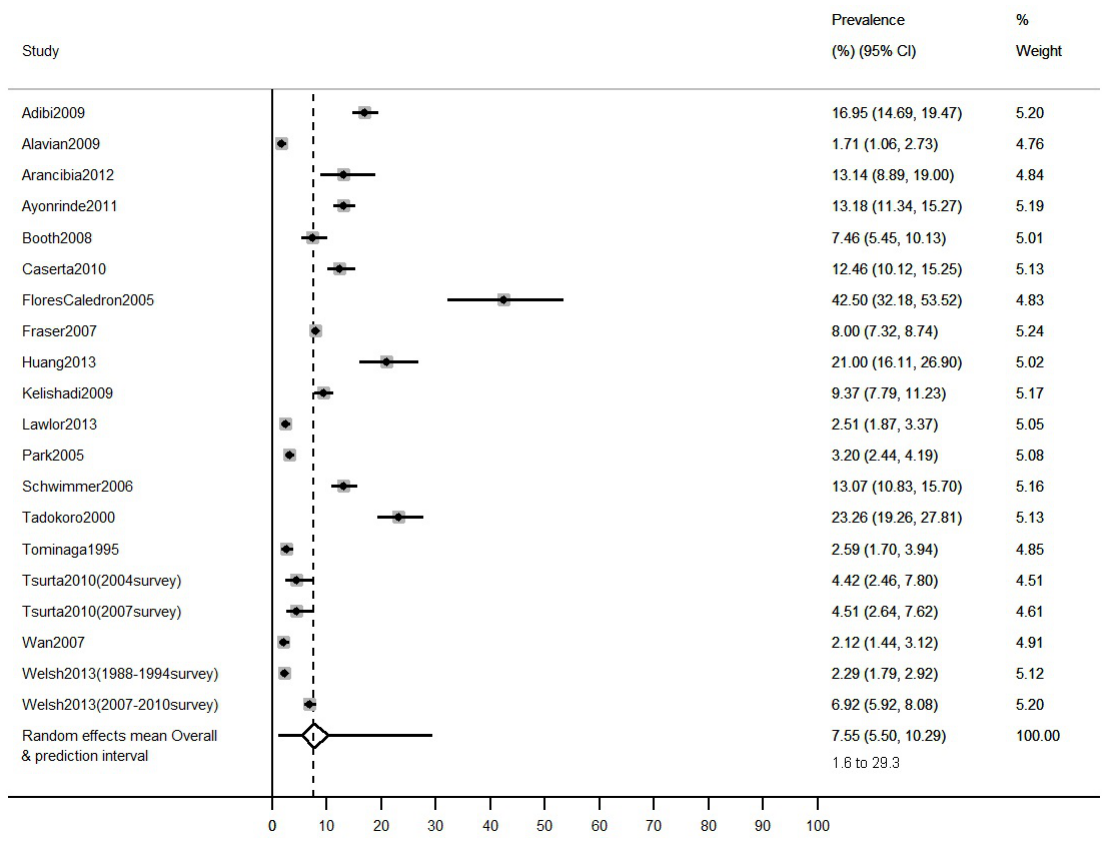
Estimates of the prevalence of NAFLD in children from general population studies. Overall mean estimate and prediction intervals are calculated from a random effects meta-analysis.

**Fig 3:**
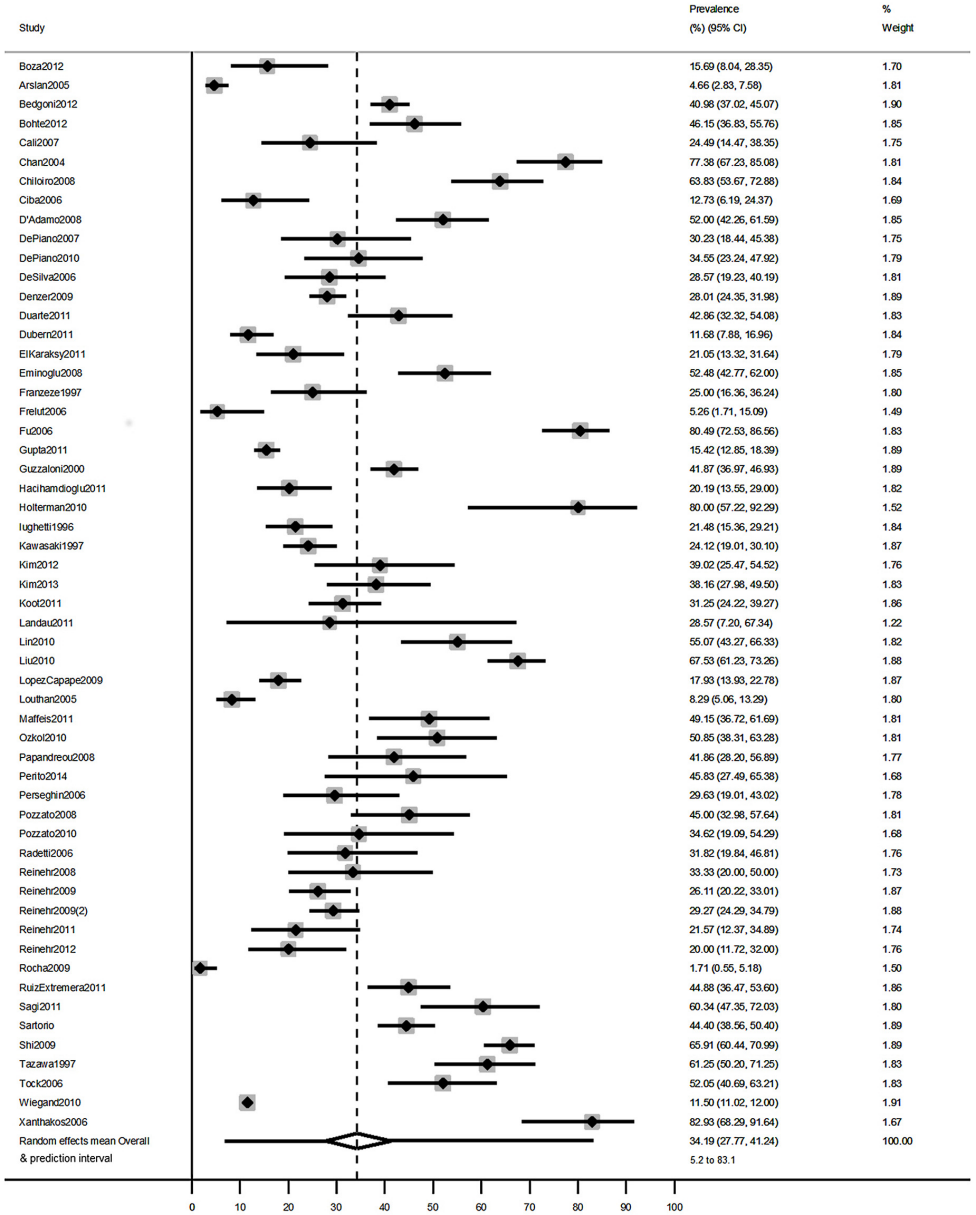
Estimates of the prevalence of NAFLD in children from clinical obese population studies. Overall mean estimate and prediction intervals are calculated from a random effects meta-analysis.

There was considerable heterogeneity among both the general population studies and the clinical obese population studies (I^2^ = 98% in both cases). The overall mean prevalence of NAFLD in general population studies was 7.6% (95%CI: 5.5% to 10.3%, N studies = 20, Fig 2). The 95% prediction interval ranged from 1.6% to 29.3%, reflecting the between-study heterogeneity. Prevalence was notably higher in studies of clinical obese populations (average across studies = 34.2%, 95%CI: 27.8% to 41.2%, N studies = 56, 95% prediction interval 5.2% to 83.1%, Fig 3).

### Differences in NAFLD prevalence by BMI, gender, age, publication year, diagnostic method, geographical region and study sample size

#### NAFLD prevalence by BMI in general population studies

Nine general population studies reported NAFLD prevalence estimates by BMI category. Meta-analysis results for each category separately (Table 1) indicate that, across studies, prevalence of NAFLD increased considerably on average with increasing BMI category. Interestingly, when stratified by diagnostic method and BMI category (Table C in S1 File), the pooled prevalence estimate from studies using USS was lower than that from studies using ALT in the normal weight group; whilst in the obese group the prevalence estimate was higher in studies using USS. The meta-analysis of within-study estimates of change in NAFLD prevalence per increase in BMI category yielded an odds ratio of 5.48 (95% CI: 3.33 to 8.99, I^2^ = 78%), which agrees with the between-study analysis (i.e. both indicate higher prevalence with higher BMI). The odds ratio for NAFLD in overweight and obese participants compared with normal weight participants was 13.36 (95% CI: 9.09 to 18.02, I^2^ = 77%) and for obese compared with normal weight and overweight participants it was 13.74 (95% CI: 9.92 to 19.03, I^2^ = 88%).

**Table 1:** NAFLD prevalence in studies reporting estimates separately for males and females, and (in general population studies only) separately for normal weight, overweight and obese subgroups.

	Prevalence (%) and 95% CI*	
	General population studies	Clinical obese population studies
**Male**	9.0 (6.5 to 12.5) (n = 15, I^2^ = 94.4, Tau2 = 0.45)	35.3 (26.0 to 45.8) (n = 27, I^2^ = 97.5, Tau2 = 1.24)
**Female**	6.3 (3.8 to 10.4) (n = 15, I^2^ = 96.5, Tau2 = 1.04)	21.8 (15.5 to 29.8) (n = 27, I^2^ = 95.7, Tau2 = 1.09)
**Normal weight**	2.3 (1.5 to 3.6) (n = 9, I^2^ = 68.9, Tau^2^ = 0.27)	-
**Overweight**	12.5 (9.2 to 16.7) (n = 9, I^2^ = 63.7, Tau^2^ = 0.12)	-
**Obese**	36.1 (24.6 to 49.4) (n = 9, I^2^ = 92.2, Tau^2^ = 0.60)	-

*Combines all diagnostic methods

#### NAFLD prevalence by gender

**Fig 4:**
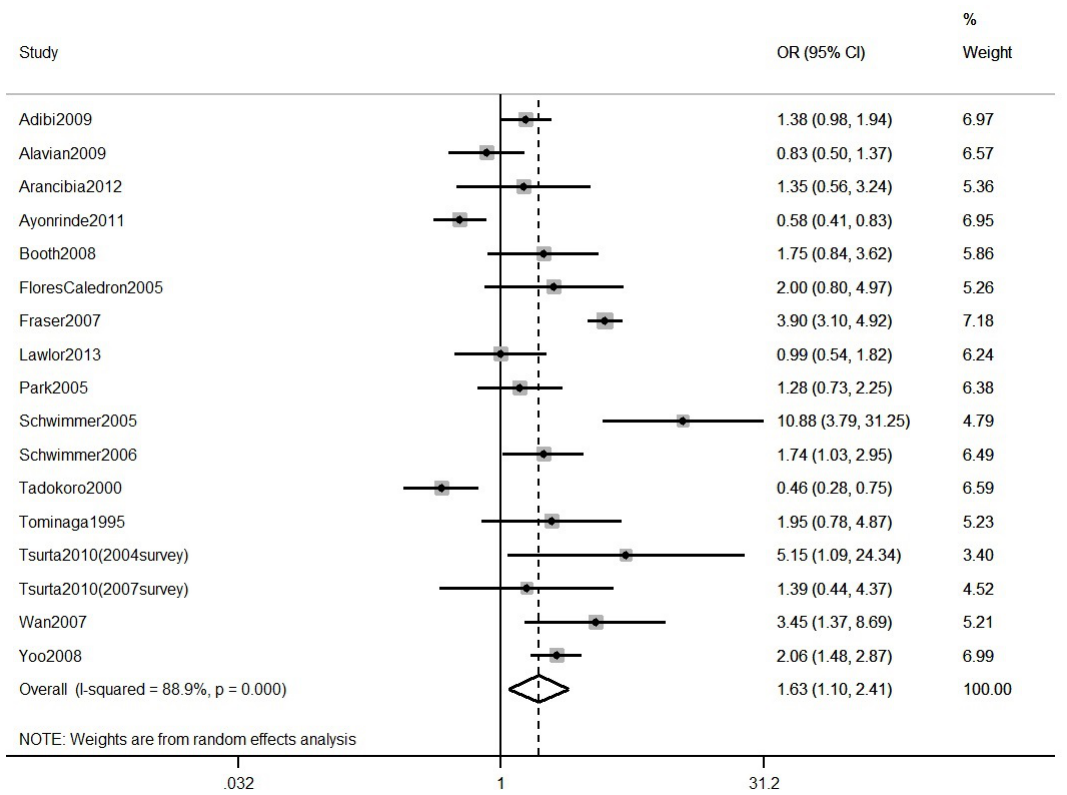
Meta-analysis of within-study comparisons of NAFLD prevalence in males versus females in general population studies.

**Fig 5:**
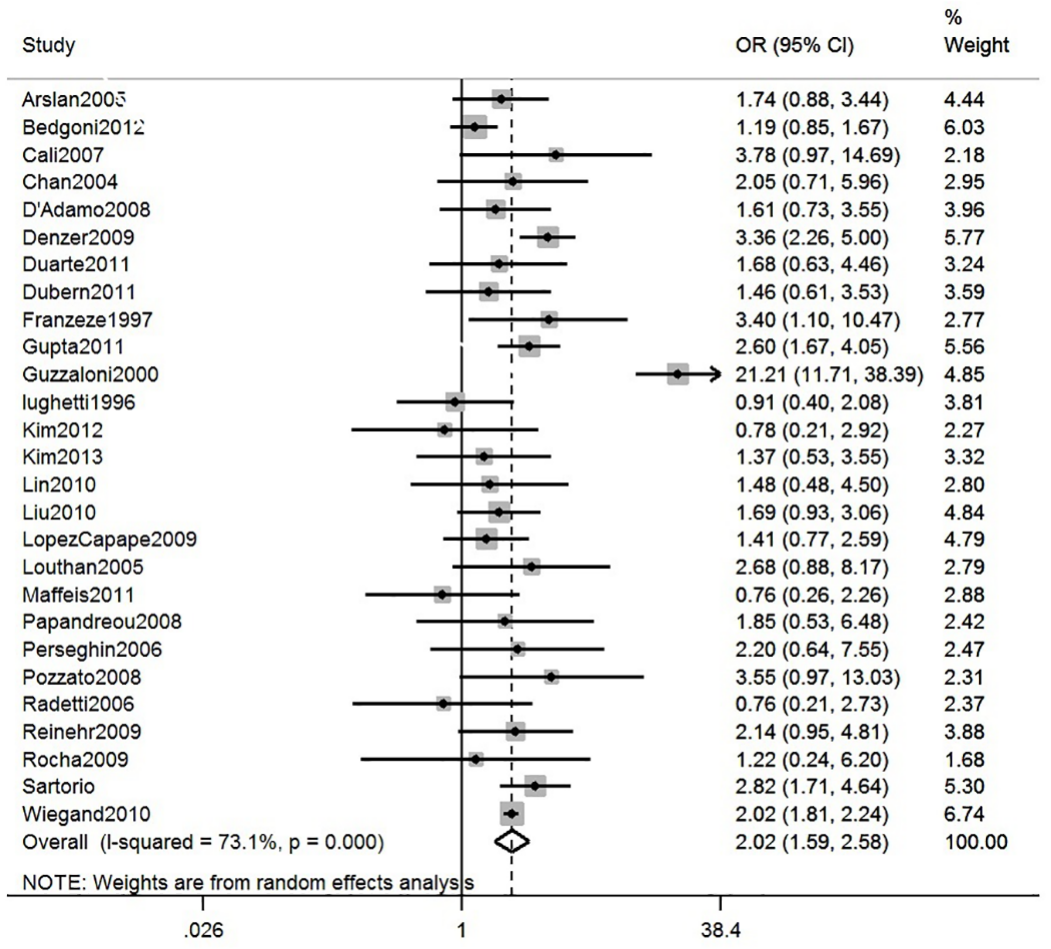
Meta-analysis of within-study comparisons of NAFLD prevalence in males versus females in clinical population studies.

15 general and 27 clinical obese population studies reported NAFLD prevalence stratified by gender. Meta-analysis of these gender-specific results (Table 1) shows that prevalence estimates were higher on average in males than females in both general population and clinical studies, although confidence intervals overlapped. Interestingly, when stratified by both diagnostic method and gender (Table D in S1 File), prevalence estimates in general population studies were similar in males and females in studies using USS, but higher in males in studies using ALT to assess NAFLD. In clinical population studies, pooled estimates were consistently higher in males, regardless of the diagnostic method used. Meta-analysis of within-study comparisons of NAFLD prevalence in males versus females in general population and clinical studies provided statistical evidence that males have higher prevalence of NAFLD than females, although with considerable heterogeneity across studies (pooled OR of males versus females in general population studies = 1.63, 95% CI: 1.10 to 2.41, I^2^ = 89%, Fig 4 and pooled OR in clinical obese studies = 2.02, 95%CI: 1.59 to 2.58, I^2^ = 73%, Fig 5).

#### NAFLD prevalence by diagnostic method

In general population studies, there was no statistical evidence from the meta-regression that NAFLD prevalence differed by diagnostic method (Table 2). In clinical studies of obese populations, there was strong statistical evidence that prevalence differed by diagnostic method. Studies using ALT had a lower mean prevalence estimate than studies using USS or MRI and in the study using biopsies prevalence was higher (Table 2).

**Table 2:** Univariable regressions of study-level predictors of NAFLD prevalence in general and clinical obese population studies.

	General population studies				Clinical population studies			
	N studies	Prevalence (95% CI)	P for difference	Residual I^2^	N studies	Prevalence (95% CI)	P for difference	Residual I^2^
**Diagnostic method**	**Total: 20**				**Total: 56**			
USS	10	7.6 (3.8 to 14.3)	0.83	97%	34	41.3 (34.6 to 48.3)	<0.01	93%
MRI	-	-			7	36.5 (18.0 to 60.1)		
ALT	9	7.0 (1.3 to 30.4)			14	13.7 (6.2 to 27.6)		
Biopsy	1	13.1 (0.7 to 76.4)			1	82.9 (36.4 to 97.6)		
**Age**	**Total: 17**				**Total: 46**			
>5 and ≤15	6	11.0 (5.5 to 20.8)	0.24	97%	25	27.5 (19.8 to 36.8)	0.27	97%
>15	9	6.4 (1.1 to 29.1)			21	36.8 (16.9 to 62.5)		
**Publication year**	**Total: 20**				**Total: 56**			
Before 2005	4	10.6 (3.7 to 26.6)	0.72	98%	8	30.4 (18.1 to 46.4)	0.34	98%
2005–2010	10	6.6 (0.6 to 44.9)			30	39.1 (11.8 to 70.9)		
After 2010	6	7.4 (0.6 to 51.2)			18	39.8 (7.7 to 63.1)		
**Geographical Region**	**Total: 20**				**Total: 56**			
Europe	2	5.7 (1.3 to 22.5)	0.50	98%	33	29.8 (23.3 to 37.2)	0.02	97%
Asia	7	5.9 (0.2 to 63.8)			7	62.3 (34.9 to 83.6)		
Middle East/North Africa	3	6.8 (0.2 to 72.5)			3	36.5 (10.6 to 73.6)		
North America	4	6.5 (0.2 to 69.1)			9	39.2 (18.1 to 65.3)		
South America	2	25.1 (0.7 to 93.7)			4	17.1 (4.9 to 45.1)		
Oceania	2	10.0 (0.3 to 82.9)			-	-		
**Study sample size** *	**Total: 20**				**Total: 56**			
≤median	10	11.2 (6.3 to 19.2)	0.06	97%	29	35.8 (27.1 to 45.7)	0.80	98%
>median	10	5.0 (1.1 to 19.6)			27	32.7 (15.3 to 56.6)		

*Median study sample size in general population studies was n = 880 and in clinical obese population studies was n = 77

All average BMIs were over 25 in clinical obese population studies

#### NAFLD prevalence by age

In both general and clinical obese population studies, there was no evidence from the meta-regression that prevalence differed in studies with an average age over 15 years compared with studies with an average age below or equal to 15 years (Table 2).

#### NAFLD prevalence by publication year

In both general and clinical obese population studies, there was no evidence from the meta-regression that prevalence differed by publication year (Table 2). The sensitivity analyses in which only studies published within the last 5 years were included in the meta-analysis provided pooled prevalence estimates of 7.1% (95% CI: 4.3% to 11.7%, N studies = 9) for general population studies and 31.8% (95% CI: 23.0% to 42.1%, N studies = 25) for clinical obese populations studies. These estimates are similar to the pooled prevalence estimates from the main analyses.

#### NAFLD prevalence by geographical region

In general population studies, there was no evidence from the meta-regression that prevalence differed by geographical region (Table 2). In clinical studies of obese populations, there was evidence that the prevalence differed by geographical region; prevalence estimates being lower in studies from South America and higher in the studies from Asia, than studies from Europe, Middle East/North Africa and North America (Table 2).

#### NAFLD prevalence by study sample size

In general population studies the meta-regression provided some evidence that prevalence was higher on average in studies with smaller sample sizes (Table 2). There was no evidence that the study sample size was associated with prevalence in clinical studies of obese populations (Table 2).

### Sensitivity analyses

For the obese clinical population studies using more than one method to estimate the prevalence of NAFLD, we randomly selected one of the methods in each study to include in analyses. Overall prevalence estimates were very similar when the alternative diagnostic method was used (33.4%, 95% CI: 27.3 to 40.2, I^2^ = 98%). Overall prevalence estimates were also similar when an arcsine transformation was used instead of logit for general population studies (8.6%, 95% CI: 6.3 to 11.3, I^2^ = 98%) and for clinical obese population studies (35.6%, 95% CI: 29.6 to 41.7, I^2^ = 98%).

## Discussion

Our systematic review, which extensively searched published evidence for all studies reporting prevalence of NAFLD in children and adolescents aged between 1 and 19 years, irrespective of whether prevalence estimate was the main aim of the study or not, has demonstrated higher NAFLD prevalences in studies in clinical obese populations (mean prevalence 34.2%) than in general population studies (7.6%). The substantial heterogeneity between studies means that the predicted range of NAFLD prevalence is wide in both clinically obese and general populations, spanning levels that are much lower than many claims in commentaries and narrative reviews [1,23] (as low as 2% and 5%, respectively in general and clinical obese populations) to very high levels which, if true, would represent a potential future major public health problem (29% and 83% respectively in general and clinically obese populations).

Meta-analysis of available within-study comparisons provided strong evidence that prevalence is higher on average in males compared with females and increases incrementally with greater BMI. However, these associations also varied considerably across studies. Meta-regression also provided some evidence of differences in prevalence across diagnostic methods and geographical areas, but residual heterogeneity from all univariable meta-regressions remained high.

Although we assessed prevalence differences by geographical region of study, we did not have sufficient information on the distribution of ethnicity in each study to assess whether NAFLD prevalence differed between ethnic groups. Furthermore, when studies used elevated ALT to diagnose NAFLD, the thresholds used ranged from 20U/L to 50U/L, with few studies using sex-specific thresholds. Studies used varying exclusion criteria to identify NAFLD cases, with many providing little or no information on this, thus, we cannot be certain that some NAFLD cases are not due to secondary causes of fatty liver, such as alcohol consumption, hepatitis or other hepatic diseases such as Wilson’s or hepatotoxic medication, and that varying exclusion criteria may explain some of the remaining heterogeneity and apparently wide prediction interval for NAFLD. That said, it is unlikely that cases are due to alcohol consumption given the average age in each of the studies (interquartile range: 11.2 to 15.0 years).

In both the clinical and general population studies, USS was the most common method for assessing NAFLD, followed by elevated ALT. In general population studies, there was no evidence that NAFLD prevalence differed by diagnostic method. In studies of clinical populations of obese children/adolescents, pooled prevalence estimates were similar when NAFLD was diagnosed by USS and MRI; however these estimates were much lower than the prevalence reported by the one clinical study using liver biopsies. This could suggest that in clinical populations of obese children/adolescents, USS and MRI underestimate NAFLD prevalence, which is plausible given the difficulty of accurate scanning in very obese individuals. However, it should also be noted that the one liver biopsy study in clinically obese young people consisted entirely of morbidly obese patients, with a mean BMI of 59.1 kg/m2 (range, 42.0–88.1 kg/m^2^) which is higher than in the other studies of clinically obese participants. Prevalence estimates in clinical studies of obese children/adolescents and in obese children/adolescents from the general population were substantially lower when elevated ALT rather than biopsies, USS or MRI was used to assess NAFLD. In contrast, in normal weight children/adolescents from the general population, NAFLD prevalence appeared to be higher when ALT was used compared to when USS was used to diagnose NAFLD. Thus, elevated ALT may underestimate NAFLD in obese young people and overestimate it in those who are normal weight.

In both general population studies and clinical studies of obese children/adolescents the prevalence was consistently higher in males than in females with all methods used to diagnose NAFLD, with the exception that in general population studies using USS to diagnose NAFLD, prevalence estimates were similar in males and females. Thus, studies should endeavour to report prevalence estimates separately for males and females.

### Study strengths and limitations

This is the first systematic review and meta-analysis of NAFLD prevalence in children and adolescents and we have systematically searched and included any study that reported a NAFLD prevalence in a paediatric or adolescent population, irrespective of the aim of the study. Although there was considerable heterogeneity between studies which was not adequately explained by a range of study characteristic, our review provides the best and most comprehensive estimate of NAFLD prevalence in young people in the general population and in clinical obese populations to date, and importantly, allows the comparison of prevalence between various groups of interest. Any systematic review and meta-analysis can only be as good as the quality of, and information in, the included studies, as well as the extent to which publication bias might influence results. Systematically searching for and including any study reporting NAFLD prevalence in a paediatric or adolescent population, irrespective of the aim of the study, may have reduced the influence of publication bias. However, there may be within-study publication bias such as not presenting prevalence stratified by gender or BMI categories when there are no differences (or differences that are in the opposite direction to those expected). Studies in which the primary aim was to examine some association (the majority of studies) might be less likely to be published if results were null (or contrary to expected) and if the likelihood of that were related to prevalence that could produce publication bias in this study. Studies used varying exclusion criteria to identify NAFLD cases and we therefore cannot rule out that some NAFLD cases are not due to secondary causes of fatty liver. The pooled prevalence estimates reflect NAFLD-spectrum disease and provides no suggestion of the severity of disease (i.e. steatosis, fibrosis or NASH). The univariable meta-regression analyses provided weak evidence of ‘small study effects’ in general population studies [30] as prevalence was on average greater in studies with smaller sample sizes.

We used random-effects meta-regressions which allow for residual heterogeneity not explained by study-level covariates.[31] However, random-effects meta-regression analyses have low power, particularly in the presence of large unexplained heterogeneity. Furthermore, between-study relationships investigated using meta-regression may not represent within-study relationships, due to the possibility of confounding (or ecological bias). Individual-level data would facilitate a more detailed examination of the dependence of NAFLD prevalence on individual-level factors and their interrelationships.

### Conclusion

The prevalence of NAFLD is higher in studies of obese clinical populations than in general population studies. Prevalence is greater in males than females and increases incrementally with BMI category. Future studies should provide detailed information on exclusion criteria used to define NAFLD. Having a standard agreed set of exclusion criteria (i.e. other potential causes of liver fat that need to be ruled out prior to arriving at a diagnosis of NAFLD) and applying these consistently in research studies and in clinical practice is imperative to understanding the true prevalence of NAFLD. Providing prevalence separately in females and males and by ethnicity may also provide a better understanding of how NAFLD prevalence varies between different populations. Finally, given that sensitivity and specificity are lower for ALT than for MRI [15], and that prevalence estimates were similar for USS and MRI, our results tentatively suggest that ALT may underestimate NAFLD prevalence in young obese people, and overestimate prevalence in the general population.

## Supporting Information

S1 Checklist (DOCX)

S1 Dataset (DTA)

S1 File (DOCX)

## Abbreviations

NAFLD: Non-alcoholic fatty liver disease
USS: Ultrasound scan
MRI: Magnetic resonance imaging
ALT: Alanine aminotransferase
AST: Aspartate aminotransferase
BMI: Body mass index
