# Supplementary material for: The Prevalence of Non-Alcoholic Fatty Liver Disease in Children and Adolescents: A Systematic Review and Meta-Analysis
Source: PLoS One. 2015 Oct 29;10(10):e0140908. doi: 10.1371/journal.pone.0140908 (PMC4626023; doi:10.1371/journal.pone.0140908)
Supplement: S1 File — (DOCX) [file pone.0140908.s003.docx]

**S1 Supporting Information**

**Protocol**

**Review title**
The prevalence of non-alcoholic fatty liver disease in children and adolescents: a systematic review and meta-analysis

**Reviewers**

Emma L Anderson^1,2^
Laura D Howe^1,2^
Debbie A Lawlor^1,2^
Abigail Fraser^1,2^.

^1^ MRC Integrative Epidemiology Unit at the University of Bristol, Bristol, UK
^2^ School of Social and Community Medicine, University of Bristol, Bristol, UK

**Centre conducting the review**

School of Social and Community Medicine, Integrative Epidemiological Unit, University of Bristol

**Review question/objective**

The quantitative objectives of this systematic review are to estimate prevalence of non-alcoholic fatty liver disease (NAFLD) in young people from (i) the general population and (ii) clinical obese populations and to determine whether prevalence estimates vary by:
-BMI category
-Gender
-Age
-Method of diagnosing NAFLD
-Geographical region
-Study sample size

**Inclusion criteria**

This review will consider studies that report prevalence of NAFLD based on any diagnostic method (i.e. biopsy, USS, MRI or other scans or liver enzymes) in participants aged between 1 and 19 years. Studies using blood-based biomarkers to estimate prevalence are eligible irrespective of the liver enzyme and threshold used to define NAFLD. Studies including participants with previous or existing liver disease (e.g. children selected for further analysis due to hepatomegaly or elevated transaminases) are not eligible for inclusion. Studies will be included if have reported a NAFLD prevalence for participants from the general population or from clinical obese populations (i.e. where participants have been recruited through their attendance at a primary or secondary care obesity service). The review will consider both experimental and epidemiological study designs including randomized controlled trials, non-randomized controlled trials, quasi-experimental, before and after studies, prospective and retrospective cohort studies, case control studies and analytical cross sectional studies, so long as the study reports a NAFLD prevalence estimate. For intervention studies the pre-intervention prevalence will be recorded.

**Search strategy**

The search strategy aims to find published studies. A two-step search strategy will be utilized in this review. A search of PubMed will be undertaken followed by analysis of the text words contained in the title and abstract, and of the index terms used to describe article. Secondly, the reference list of all identified reports and articles will be searched for additional studies. Studies published in non-English languages will be considered for inclusion in this review. Studies published up to October 2013 will be considered for inclusion in this review. The search will be restricted to humans.

The PubMed search terms are as follows:

((Liver enzymes) OR (Alanine aminotransferase) OR (Alanine transaminase) OR (Gamma-glutamyl transpeptidase) OR (Gamma-glutamyl transferase) OR (Gamma-glutamyltransferase) OR (Aspartate aminotransferases) OR (Aspartate transaminase)) OR ((Non-alcoholic fatty liver) OR (Nonalcoholic fatty liver) OR (NAFLD) OR (Non-alcoholic steatohepatitis) OR (Nonalcoholic steatohepatitis) OR (Non-alcoholic steatosis) OR (Nonalcoholic steatosis) OR (Steatosis) OR (Fatty liver) OR (hepatic steatosis) OR (hepatic fat)) AND ((child) OR (children) OR (paediatric) OR (pediatric) OR (adolescen*) OR (infant[MeSH] OR child[MeSH] OR adolescent[MeSH]))

**Data extraction**

A standardised proforma will be created for the extraction of relevant information from identified studies and contents of this proforma will be agreed between Emma Anderson (EA), Debbie Lawlor (DL) Abigail Fraser (AF) and Laura Howe (LH). EA will review all titles retrieved by the PubMed search, and will exclude any clearly irrelevant publications. EA will then screen all remaining abstracts for eligibility. Full text papers for all potentially eligible studies will be obtained and will be further evaluated by EA. Papers will be discarded if they present no original data. If two studies are published on the same cohort of participants, the largest study will be included in this review. Studies not accessible from any British Library will be excluded. Studies that we are unable to translate to English will also be excluded. Data for all relevant papers will be extracted by EA. Data will also be extracted by one of DL, LH or AF, so that two investigators will review each paper independently using the standardised proforma. Any discrepancies in the extractions will be discussed and an agreement will be made by consensus between EA, DL, AF and LH. Data from non-English language papers will be extracted by colleagues who are fluent in the relevant language. The prevalence of NAFLD will be extracted for the overall study sample and also separately by gender and BMI category (i.e. normal weight, overweight and obese). Study authors will be contacted for additional data or clarification as required.

**Data synthesis**

EA will conduct meta-analyses in Stata MP to examine NAFLD prevalence in the general population and for clinical obese populations. For studies using more than one method to estimate the prevalence of NAFLD (for example, studies reporting two separate NAFLD prevalence estimates by USS and by elevated ALT), one of the methods will be randomly selected to be included in analyses. For any studies comparing NAFLD prevalence in obese participants with prevalence in matched non-obese participants (e.g. matched on age and gender), the non-obese group will be excluded from analyses as the matching process may result in a sample that is not representative of the general population. Meta-analyses will be performed for each gender and BMI subgroups to provide estimates of prevalence for each gender or BMI category. Univariable meta-regression will be used to assess whether the following study-level characteristics are associated with NAFLD prevalence in both general and clinical population studies: diagnostic method, average age of participants, country of study and sample size.

**Conflicts of interest**

None of the authors of this systematic review have any conflicts of interests to declare.

**PRISMA checklist**

|  |  |  |  |
| --- | --- | --- | --- |
| Section/topic | Item No | Checklist item | Reported on page No |
| **Title** | | | |
| Title | 1 | Identify the report as a systematic review, meta-analysis, or both | Page 1, line 1 |
| **Abstract** | | | |
| Structured summary | 2 | Provide a structured summary including, as applicable, background, objectives, data sources, study eligibility criteria, participants, interventions, study appraisal and synthesis methods, results, limitations, conclusions and implications of key findings, systematic review registration number | Page 4 |
| **Introduction** | | | |
| Rationale | 3 | Describe the rationale for the review in the context of what is already known | Page 5, lines 81-129 |
| Objectives | 4 | Provide an explicit statement of questions being addressed with reference to participants, interventions, comparisons, outcomes, and study design (PICOS) | Page 5, line 129 to page 6, line 131 |
| **Methods** | | | |
| Protocol and registration | 5 | Indicate if a review protocol exists, if and where it can be accessed (such as web address), and, if available, provide registration information including registration number | Not applicable |
| Eligibility criteria | 6 | Specify study characteristics (such as PICOS, length of follow-up) and report characteristics (such as years considered, language, publication status) used as criteria for eligibility, giving rationale | Page 7, line 133 |
| Information sources | 7 | Describe all information sources (such as databases with dates of coverage, contact with study authors to identify additional studies) in the search and date last searched | Page 7, line 141 |
| Search | 8 | Present full electronic search strategy for at least one database, including any limits used, such that it could be repeated | Page 7, line 145 |
| Study selection | 9 | State the process for selecting studies (that is, screening, eligibility, included in systematic review, and, if applicable, included in the meta-analysis) | Page 7, line 149 |
| Data collection process | 10 | Describe method of data extraction from reports (such as piloted forms, independently, in duplicate) and any processes for obtaining and confirming data from investigators | Page 8, line 155 |
| Data items | 11 | List and define all variables for which data were sought (such as PICOS, funding sources) and any assumptions and simplifications made | Page 8, line 155 |
| Risk of bias in individual studies | 12 | Describe methods used for assessing risk of bias of individual studies (including specification of whether this was done at the study or outcome level), and how this information is to be used in any data synthesis | Page 19, line 432- 453 |
| Summary measures | 13 | State the principal summary measures (such as risk ratio, difference in means). | Page 8, line 161 |
| Synthesis of results | 14 | Describe the methods of handling data and combining results of studies, if done, including measures of consistency (such as I^2^ statistic) for each meta-analysis | Page 8, line 169 to page 10, line 229 |
| Risk of bias across studies | 15 | Specify any assessment of risk of bias that may affect the cumulative evidence (such as publication bias, selective reporting within studies) | Page 19, line 432- 453 |
| Additional analyses | 16 | Describe methods of additional analyses (such as sensitivity or subgroup analyses, meta-regression), if done, indicating which were pre-specified | Page 14, line 321 |
| **Results** | | | |
| Study selection | 17 | Give numbers of studies screened, assessed for eligibility, and included in the review, with reasons for exclusions at each stage, ideally with a flow diagram | Page 11, line 231 |
| Study characteristics | 18 | For each study, present characteristics for which data were extracted (such as study size, PICOS, follow-up period) and provide the citations | Page 11, line 232 |
| Risk of bias within studies | 19 | Present data on risk of bias of each study and, if available, any outcome-level assessment (see item 12). | Page 19, line 432- 453 |
| Results of individual studies | 20 | For all outcomes considered (benefits or harms), present for each study (a) simple summary data for each intervention group and (b) effect estimates and confidence intervals, ideally with a forest plot | Page 11, line 232 |
| Synthesis of results | 21 | Present results of each meta-analysis done, including confidence intervals and measures of consistency | Page 11, line 253 |
| Risk of bias across studies | 22 | Present results of any assessment of risk of bias across studies (see item 15) | Page 19, line 432- 453 |
| Additional analysis | 23 | Give results of additional analyses, if done (such as sensitivity or subgroup analyses, meta-regression) (see item 16) | Page 14, line 321 |
| **Discussion** | | | |
| Summary of evidence | 24 | Summarise the main findings including the strength of evidence for each main outcome; consider their relevance to key groups (such as health care providers, users, and policy makers) | Page 15, line 331 |
| Limitations | 25 | Discuss limitations at study and outcome level (such as risk of bias), and at review level (such as incomplete retrieval of identified research, reporting bias) | Page 17, line 381 |
| Conclusions | 26 | Provide a general interpretation of the results in the context of other evidence, and implications for future research | Page 18, line 427 |
| **Funding** | | | |
| Funding | 27 | Describe sources of funding for the systematic review and other support (such as supply of data) and role of funders for the systematic review | Page 2, line 25 |

**Search terms**The following search terms were entered into PubMed: ((Liver enzymes) OR (Alanine aminotransferase) OR (Alanine transaminase) OR (Gamma-glutamyl transpeptidase) OR (Gamma-glutamyl transferase) OR (Gamma-glutamyltransferase) OR (Aspartate aminotransferases) OR (Aspartate transaminase)) OR ((Non-alcoholic fatty liver) OR (Nonalcoholic fatty liver) OR (NAFLD) OR (Non-alcoholic steatohepatitis) OR (Nonalcoholic steatohepatitis) OR (Non-alcoholic steatosis) OR (Nonalcoholic steatosis) OR (Steatosis) OR (Fatty liver) OR (hepatic steatosis) OR (hepatic fat)) AND ((child) OR (children) OR (paediatric) OR (pediatric) OR (adolescen*) OR (infant[MeSH] OR child[MeSH] OR adolescent[MeSH])).

**Table A: Summary of general population studies included in the systematic review and meta-analysis**

| **Author, year, participant group, reference** | **Country** | **Total N  (% male)** | **Age** | **Scan type or biopsy measure of NAFLD** | **Enzymes and thresholds used to indicate NAFLD** | **Prevalence reported by gender** | **Prevalence reported by BMI category** |
| --- | --- | --- | --- | --- | --- | --- | --- |
| Adibi, 2009(1) | Iran | 950 (44) | Range: 6 to 19 | USS |  | Y | Y |
| Alavian, 2009(2) | Iran | 966 (45) | Range: 7 to 18 | USS | ALT>40U/L | Y | Y |
| Arancibia, 2012(3) | Chile | 175 (46) | Range: 9 to 14 |  | ALT>41U/L in males and >33 in females | Y | N |
| Ayonrinde, 2011(4) | Australia | 1138 (52) | Mean: 17.0; SD: 0.25 | USS |  | Y | N |
| Booth, 2008(5) | Australia | 496 (59) | Range: 14 to 17 |  | ALT = 32U/L and 20U/L for boys and girls respectively. | Y | Y |
| Caserta, 2010(6) | Italy | 642(49) | Range: 11 to 13 | USS |  | N | N |
| Flores-Caledron, 2005(8) | Mexico | 80 (55) | Mean: 9.5; SD: 1.15 |  | ALT >40U/l | Y | N |
| Fraser, 2007(9) | USA | 5586 (50) | Range: 12 to 19 |  | ALT >30U/L | Y | N |
| Huang, 2013(12) | Taiwan | 219 (64) | Range: 6 to 12 | USS |  | N | Y |
| Kelishadi, 2009(13) | Iran | 1110 (44) | Mean: 12.57; SD: 3.3 |  | ALT >90th percentile for the study sample | N | Y |
| Lawlor, 2013(15) | United Kingdom | 1723 (41) | Mean:17.94; SD:0.44 | USS |  | Y | N |
| Park, 2005(25) | Korea | 1594 (97) | Range: 10 to 19 |  | ALT >40U/L | Y | N |
| Schwimmer, 2006(29) | USA | 742 (72) | Range: 2 to 19 | Biopsy |  | Y | Y |
| Tadokoro, 2000(30) | Japan | 374 (52) | Mean: 15.4; SD: 0.03 | USS |  | Y | N |
| Tominaga, 1995(31) | Japan | 810 (51) | Range: 2 to 12 | USS |  | Y | Y |
| Tsuruta, 2010 (2004 survey)(32) | Japan | 249 (48) | Junior high school students | USS |  | Y | Y |
| Tsuruta, 2010 (2007 survey) (33) |  | 288 (54) |  |  |  |  |  |
| Wan, 2007(34) | China | 1180 (49) | Range: 6 to 14 | USS |  | Y | N |
| Welsh, 2013 (NHANES 1988-1994 survey)(35) | USA | 2748(51) | Mean: 15.4; SE: 0.1 |  | ALT>30U/L and >40U/L | N | N |
| Welsh, 2013 (NHANES 2007-2010 survey)(35) |  | 2138(52) | Mean: 15.5; SE: 0.1 |  |  |  |  |

SD= standard deviation, IQR=interquartile range

**Table B:** **Summary of clinical studies of obese children/adolescents included in the systematic review and meta-analysis**

| **Author, year, reference** | **Country** | **Total N (% male)** | **Age** | **Definition of obesity** | **Scan type or biopsy measure of NAFLD** | **Enzymes and thresholds used to define elevated biomarkers** | **Prevalence reported by gender** |
| --- | --- | --- | --- | --- | --- | --- | --- |
| Arslan, 2005(39) | Turkey | 322 (43) | Range: 4 to 18 | BMI of the patients were proportioned to the BMI of age- and sex matched children in the 50th percentile, then multiplied by 100. A ratio above 120 was accepted as obesity | USS | ALT >45 U/L | Y |
| Bedgoni, 2012(40) | Italy | 571 (57) | Range: 8 to18 | BMI above the 95^th^ percentile | USS |  | Y |
| Bohte, 2012(41) | Holland | 104 (45) | Range: 8 to 19 | BMI above the 95^th^ percentile | MRI |  |  |
| Boza, 2012(42) | Chile | 51 (20) | Range: 15 to 19 | Either BMI > 40 mg/kg2, or BMI > 35 mg/kg2 with associated co-morbidities | USS |  |  |
| Cali, 2007(43) | USA | 49(35) | Mean: 15.3; SD: 2.3 | BMI above the 95^th^ percentile | MRI |  | Y |
| Chan, 2004(44) | China | 84 (70) | Range: 7 to 18 | BMI above the 95^th^ percentile | USS |  | Y |
| Chiloiro, 2008(45) | Italy | 94 (63) | Mean: 9.7; SD 2.2 | BMI z-score for age, sex and ethnicity >2 | USS |  | N |
| Ciba, 2007(46) | Austria | 55 | Mean: 11.7; SD: 3.5 | BMI above the 97^th^ percentile | USS | ALT >40U/l | N |
| D'Adamo, 2008(47) | Italy | 100 (52) | Mean: 8.5; SD: 1.89 | BMI above the 98^th^ percentile | USS |  | Y |
| De Piano, 2007(48) | Brazil | 43 (44) | Range: 15 to 19 | BMI > 30 mg/kg2 | USS |  | N |
| De Piano, 2010(49) | Brazil | 55 (46) | Range: 15 to 19 | BMI above the 95^th^ percentile | USS |  | N |
| de Silva, 2006(50) | Sri Lanka | 70(57) | Range: 2.1 to 14.75 | BMI above the 95^th^ percentile | USS |  | N |
| Denzer, 2009(51) | Germany | 532 (45) | Range: 8 to 19 | BMI above the 90^th^ percentile | USS |  | Y |
| Duarte, 2011(52) | Brazil | 77 (66) | Range: 2 to 14 | BMI above the 95^th^ percentile | USS |  | Y |
| Dubern, 2006(53) | France | 197 (36) | Mean: 11.4; SD: 3.0 | BMI above the 99.8^th^ percentile |  | ALT >35 U/L | Y |
| El-karaksy, 2011(54) | Egypt | 76 (50) | Range: 2 to 15 | BMI above the 85^th^ percentile | USS | ALT >41U/L | N |
| Eminoglu, 2008(55) | Turkey | 101 (49) | Median: 10.6; IQR: 7.6 to 13.6 | BMI above the 95^th^ percentile | USS | ALT above normal range for this sample | Y |
| Franzeze, 1997(56) | Italy | 72 (44) | Range: 4.5 to 16 | Percentage of ideal body weight for height, calculated on NCHS tables adapted in reference to the regional population standard. An ideal body weight higher than 160% was considered as morbid obesity. All patients had an IBW over 120%. | USS |  | Y |
| Frelut, 2006(57) | France | 57 | Mean: 14.1; SD: 1.5 | Not given |  | ALT>50U/L | N |
| Fu, 2006(58) | China | 123 (71) | Range: 7 to 16 | BMI >23 mg/kg2 | USS | ALT (threshold not listed) |  |
| Gupta, 2011(59) | USA | 655 (45.64) | Mean: 11.9; SD: 3.5 | BMI above the 95^th^ percentile |  | ALT >40U/L | Y |
| Guzzaloni, 2000(60) | Italy | 375 (55) | Mean: 13.8; SD: 1.9 | BMI above the 98^th^ percentile | USS | ALT>40U/L | Y |
| Hacihamdioglu, 2011 (61) | Turkey | 104 (38) | Mean: 9.3; SD: 2.5 | BMI above the 95^th^ percentile | USS |  | N |
| Holterman, 2010(62) | USA | 20 (25) | Mean: 16.0; SD: 1.0 | Not given | USS |  | N |
| Iughetti, 1996(63) | Italy | 135 (57) | Range: 4 to 16 | Not given | USS |  | Y |
| Kawasaki, 1997(64) | Japan | 228 (61) | Range: 6.5 to 15.6 | Percent obesity calculated as (body weight - ideal body weight) ÷ ideal body weight × 100. Subjects whose percent obesity ranged from 20 to 29% and from 30 to 49%, and reached 50% or more, were estimated to be mildly, moderately, and severely obese, respectively |  | ALT >35U/L | N |
| Kim, 2012(65) | USA | 41 (66) | Range: 14 to 17 | BMI above the 85^th^ percentile | MRI |  | Y |
| Kim, 2013(66) | USA | 76 (37) | Mean: 13.6; SD: 2.6 | Not given | MRI |  | Y |
| Koot, 2011(67) | Holland | 144 (37.5) | Mean: 14.1; SD: 2.3 | BMI for age >35 kg/m2 | USS |  | N |
| Landau 2011(68) | Israel | 7 | Range: 13.8 to 18 | Not given | USS |  | N |
| Lin, 2010(69) | Taiwan | 69 (77) | Range: 6.3 to 17.6 | BMI above the 95^th^ percentile | USS | ALT > 0.50U/L for boys and >0.32U/L for girls | Y |
| Liu, 2010(70) | China | 231 (71) | Range: 7 to 14 | BMI above the 95^th^ percentile | USS |  | Y |
| Lopez-Capape, 2009(71) | Spain | 290 (51) | Range: 4-18 | BMI above the 98^th^ percentile | USS | ALT, AST or GGT >40U/L | Y |
| Louthan, 2005(72) | USA | 181 (45) | Range: 4 to 17.9 | BMI above the 95^th^ percentile |  | ALT >40 U/L | Y |
| Maffeis, 2011(73) | Italy | 59 (36) | Mean: 10.5; SD: 1.95 | BMI above the 95^th^ percentile | MRI |  | Y |
| Ozkol, 2010(74) | Turkey | 59 (41) | Range: 9.1 to 18.0 | BMI above the 95^th^ percentile | USS |  | N |
| Papandreou, 2008(75) | Greece | 43 (58) | Range: 9 to 14 | BMI above the 95^th^ percentile | USS |  | Y |
| Perito, 2014(76) | USA | 24 | Mean: 11.6; SD: 2.9 | BMI above the 85^th^ percentile | USS | ALT >50 U/L | N |
| Perseghin, 2006(77) | Italy | 54 (56) | Range: 11 to 18 | BMI above the 99^th^ percentile | MRI |  | Y |
| Pozzato, 2008(78) | Italy | 60 (48) | Range: 6 to 14 | BMI > age- and sex-adjusted curve passing through the cut-off point of 30 kg/m2 at 18 years | USS and MRI |  | Y |
| Pozzato, 2010(79) | Italy | 26 (42) | Range: 6 to 14 | BMI > age- and sex-adjusted curve passing through the cut-off point of 30 kg/m2 at 18 years | MRI |  | N |
| Radetti, 2006(80) | Italy | 44 (54) | Range: 6 to 16 | BMI above the 97^th^ percentile | MRI | ALT>40 U/L | Y |
| Reinehr, 2008(81) | Germany | 36 (44) | Mean: 10.53; SD: 2.29 | Obesity was defined according to the definition of the International Task Force of Obesity using population-specific data | USS |  | N |
| Reinehr, 2009(82) | Germany | 180 (48) | Mean: 10.7; SD: 2.5 | Obesity was defined according to the definition of the International Task Force of Obesity using population-specific data | USS | ALT >20U/L | Y |
| Reinehr, 2009(83) | Germany | 287 (47) | Range: 4 to 16 | BMI above the 97^th^ percentile | USS |  | N |
| Reinehr, 2011(84) | Germany | 51 (55) | Mean: 12.1; SD: 2.3 | BMI above the 97^th^ percentile | USS |  | N |
| Reinehr, 2012(85) | Germany | 60 | Mean: 12.0; SD: 1.4 | Obesity was defined according to the definition of the International Task Force of Obesity using population-specific data | USS |  | N |
| Rocha, 2009(86) | Brazil | 175 (46) | Range: 11 to 18 | BMI above the 85^th^ percentile | USS | ALT >52 U/L for girls and >72 U/L for boys | Y |
| Ruiz-extremera, 2011(87) | Spain | 127 (50) | Range: 4 to 15 | BMI above the 95^th^ percentile | USS |  | N |
| Sagi, 2007(88) | Israel | 58 (50) | Mean: 14.35; SD: 2.4 | BMI above the 95^th^ percentile | USS | ALT ≥40 U/L | N |
| Sartorio, 2007(89) | Italy | 268 (44) | Mean: 14.77; SD: 3.39 | BMI above the 90^th^ percentile | USS |  | Y |
| Shi, 2009(90) | China | 308 (75) | Range: 9 to 14 | BMI above the 95^th^ percentile | USS |  | N |
| Tazawa, 1997(91) | Japan | 80 | Range: 6 to 14 | Obesity was defined as the degree of obesity which was more than 20% higher than that of the ideal weight. | USS |  | N |
| Tock, 2006(92) | Brazil | 73 (33) | Range: 15 to 19 | BMI above the 95^th^ percentile | USS |  | N |
| Wiegand, 2010(93) | Germany, Austria and Switzerland | 16390 (58) | Median:12.5; IQR: 10.8 to 14.6 | BMI above the 90^th^ percentile |  | ALT>50 U/L | Y |
| Xanthakos, 2006(94) | USA | 41 (39) | Range: 13 to 19 | Not given | Biopsy |  | N |

SD= standard deviation, IQR=interquartile range

**Table C:** **NAFLD prevalence in the general population studies reporting prevalence estimates separately for normal weight, overweight and obese subgroups, when stratified by diagnostic method**

|  | **USS** | **ALT** |
| --- | --- | --- |
| **Normal weight** | 1.5 (0.8 to 2.7)  (n=6, I^2^=56.5, Tau^2^=1.31) | 3.6 (2.5 to 5.1)  (n=2, I^2^=0.0, Tau^2^=0.0) |
| **Overweight** | 12.1 (8.2 to 17.5) (n=6, I^2^=58.1, Tau^2^=0.13) | 13.5 (6.5 to 26.2)  (n=2, I^2^=85.7, Tau^2^=0.30) |
| **Obese** | 41.6 (31.5 to 27.1)  (n=6, I^2^=88.0, Tau^2^=0.55) | 20.3 (12.1 to 32.2)  (n=2, I^2^=56.0, Tau^2^=0.13) |

**Table D: NAFLD prevalence in general population studies and clinical obese population studies reporting prevalence estimates separately for males and females, when stratified by diagnostic method**

|  | **General population studies** | |  | **Clinical obese population studies** | |
| --- | --- | --- | --- | --- | --- |
|  | **Male** | **Female** |  | **Male** | **Female** |
| **MRI** | No studies | No studies |  | 37.0 (30.2 to 44.4)  (n=7, I^2^=0.0, Tau^2^=0.0) | 29.2 (20.3 to 40.0) (n=7, I^2^=55.5, Tau^2^=0.23) |
| **USS** | 6.8 (4.0 to 11.3) (n=9, I^2^=94.2, Tau^2^=0.66) | 5.5 (2.8 to 10.6)  (n=9, I^2^=96.5, Tau^2^=1.04) |  | 41.9 (31.3 to 53.2) (n=15, I^2^=94.6, Tau^2^=0.75) | 27.9 (19.8 to 37.8)  (n=16, I^2^=91.3, Tau^2^=0.67) |
| **ALT** | 13.1 (6.6 to 24.5) (n=5, I^2^=95.8, Tau^2^=0.71) | 7.4 (3.4 to 15.4) (n=5, I^2^=94.0, Tau^2^=0.81) |  | 19.4 (12.6 to 28.6) (n=7, I^2^=91.6, Tau^2^=0.38) | 9.5 (6.6 to 13.5) (n=8, I^2^=76.1, Tau^2^=0.19) |

Reference List

1. Adibi A, Kelishadi R, Beihaghi A, Salehi H, Talaei M. Sonographic fatty liver in overweight and obese children, a cross sectional study in Isfahan. Endokrynologia Polska 2009;2009/02/19:14-9.

2. Alavian SM, Mohammad-Alizadeh AH, Esna-Ashari F, Ardalan G, Hajarizadeh B. Non-alcoholic fatty liver disease prevalence among school-aged children and adolescents in Iran and its association with biochemical and anthropometric measures. Liver international : official journal of the International Association for the Study of the Liver 2009;2008/05/22:159-63.

3. Arancibia G, Garcia H, Jaime F, Bancalari R, Harris PR. [Association of metabolic syndrome markers with abnormal alanine aminotransferase levels in healthy children]. Revista medica de Chile 2012;140:896-901.

4. Ayonrinde OT, Olynyk JK, Beilin LJ et al. Gender-specific differences in adipose distribution and adipocytokines influence adolescent nonalcoholic fatty liver disease. 2011;53:800-9.

5. Booth ML, George J, Denney-Wilson E et al. The population prevalence of adverse concentrations and associations with adiposity of liver tests among Australian adolescents. 2008;44:686-91.

6. Caserta CA, Pendino GM, Amante A et al. Cardiovascular risk factors, nonalcoholic fatty liver disease, and carotid artery intima-media thickness in an adolescent population in southern Italy. Am J Epidemiol 2010;2010/05/12:1195-202.

7. Suano de Souza FI, Silverio Amancio OM, Saccardo Sarni RO et al. Non-alcoholic fatty liver disease in overweight children and its relationship with retinol serum levels. International journal for vitamin and nutrition research Internationale Zeitschrift fur Vitamin- und Ernahrungsforschung Journal international de vitaminologie et de nutrition 2008;78:27-32.

8. Flores-Calderon J, Gomez-Diaz RA, Rodriguez-Gomez G, Moran-Villota S. Frequency of increased aminotransferases levels and associated metabolic abnormalities in obese and overweight children of an elementary school in Mexico City. Annals of hepatology 2005;2006/01/25:279-83.

9. Fraser A, Longnecker MP, Lawlor DA. Prevalence of Elevated Alanine Aminotransferase Among US Adolescents and Associated Factors: NHANES 1999-2004. Gastroenterology 2007;133:1814-20.

10. Fu CC, Chen MC, Li YM, Liu TT, Wang LY. The risk factors for ultrasound-diagnosed non-alcoholic fatty liver disease among adolescents. Annals of the Academy of Medicine, Singapore 2009;2009/02/18:15-7.

11. Gronbaek H, Lange A, Birkebaek NH et al. Effect of a 10-week weight loss camp on fatty liver disease and insulin sensitivity in obese Danish children. J Pediatr Gastroenterol Nutr 2012;54:223-8.

12. Huang SC, Yang YJ. Serum retinol-binding protein 4 is independently associated with pediatric NAFLD and fasting triglyceride level. Journal of Pediatric Gastroenterology and Nutrition 2013;56:145-50.

13. Kelishadi R, Cook SR, Adibi A et al. Association of the components of the metabolic syndrome with non-alcoholic fatty liver disease among normal-weight, overweight and obese children and adolescents. Diabetology & metabolic syndrome 2009;2009/12/24:29.

14. Kim IK, Kim J, Kang JH, Song J. Serum leptin as a predictor of fatty liver in 7-year-old Korean children. Annals of nutrition & metabolism 2008;2008/10/24:109-16.

15. Lawlor DA, Callaway M, Macdonald-Wallis C et al. Nonalcoholic Fatty Liver Disease, Liver Fibrosis, and Cardiometabolic Risk Factors in Adolescence: A Cross-Sectional Study of 1874 General Population Adolescents. J Clin Endocrinol Metab 2014;jc20133612.

16. Lin YC, Chang PF, Hu FC, Chang MH, Ni YH. Variants in the UGT1A1 gene and the risk of pediatric nonalcoholic fatty liver disease. Pediatrics 2009;2009/12/02:e1221-e1227.

17. Lin YC, Chang PF, Hu FC, Yang WS, Chang MH, Ni YH. A common variant in the PNPLA3 gene is a risk factor for non-alcoholic fatty liver disease in obese Taiwanese children. The Journal of Pediatrics 2011;2010/12/21:740-4.

18. Lin YC, Chang PF, Chang MH, Ni YH. A common variant in the peroxisome proliferator-activated receptor-gamma coactivator-1alpha gene is associated with nonalcoholic fatty liver disease in obese children. The American Journal of Clinical Nutrition 2013;97:326-31.

19. Lira AR, Oliveira FL, Escrivao MA, Colugnati FA, Taddei JA. Hepatic steatosis in a school population of overweight and obese adolescents. Jornal de pediatria 2010;2010/02/13:45-52.

20. Monteiro PA, Mota J, Silveira LS et al. Morphological and metabolic determinants of nonalcoholic fatty liver disease in obese youth: a pilot study. BMC research notes 2013;6:89.

21. Okamatsu Y, Matsuda K, Hiramoto I et al. Ghrelin and leptin modulate immunity and liver function in overweight children. Pediatrics international : official journal of the Japan Pediatric Society 2009;2009/04/18:9-13.

22. Oliveira AC, Oliveira AM, Almeida MS, Silva AM, Adan L, Ladeia AM. Alanine aminotransferase and high sensitivity C-reactive protein: correlates of cardiovascular risk factors in youth. The Journal of Pediatrics 2008;152:337-42.

23. Oliveira AM, Oliveira N, Reis JC, Santos MV, Silva AM, Adan L. Triglycerides and alanine aminotransferase as screening markers for suspected fatty liver disease in obese children and adolescents. Hormone research 2009;2009/01/09:83-8.

24. Papandreou D, Karabouta Z, Pantoleon A, Rousso I. Investigation of anthropometric, biochemical and dietary parameters of obese children with and without non-alcoholic fatty liver disease. Appetite 2012;59:939-44.

25. Park HS, Han JH, Choi KM, Kim SM. Relation between elevated serum alanine aminotransferase and metabolic syndrome in Korean adolescents. The American Journal of Clinical Nutrition 2005;82:1046-51.

26. Quiros-Tejeira RE, Rivera CA, Ziba TT, Mehta N, Smith CW, Butte NF. Risk for nonalcoholic fatty liver disease in Hispanic youth with BMI > or =95th percentile. J Pediatr Gastroenterol Nutr 2007;44:228-36.

27. Lockitch G, Halstead AC, Albersheim S, MacCallum C, Quigley G. Age- and sex-specific pediatric reference intervals for biochemistry analytes as measured with the Ektachem-700 analyzer. Clin Chem 1988;34:1622-5.

28. Schwimmer JB, McGreal N, Deutsch R, Finegold MJ, Lavine JE. Influence of Gender, Race, and Ethnicity on Suspected Fatty Liver in Obese Adolescents. Pediatrics 2005;115:e561-e565.

29. Schwimmer JB, Deutsch R, Kahen T, Lavine JE, Stanley C, Behling C. Prevalence of Fatty Liver in Children and Adolescents. Pediatrics 2006;118:1388-93.

30. Tadokoro N, Shinomiya M, Yoshinaga M et al. Visceral fat accumulation in Japanese high school students and related atherosclerotic risk factors. Journal of atherosclerosis and thrombosis 2010;2010/06/22:546-57.

31. Tominaga K, Kurata J, Chen Y et al. Prevalence of fatty liver in Japanese children and relationship to obesity. Digestive Diseases and Sciences 1995;40:2002-9.

32. Tsuruta G, Tanaka N, Hongo M et al. Nonalcoholic fatty liver disease in Japanese junior high school students: its prevalence and relationship to lifestyle habits. J Gastroenterol 2010;45:666-72.

33. Tsuruta G, Tanaka N, Hongo M et al. Nonalcoholic fatty liver disease in Japanese junior high school students: its prevalence and relationship to lifestyle habits. Journal of gastroenterology 2010;2010/01/20:666-72.

34. Wan YP, Xu RY, Fang H, Lu LP, Zhang XM, Cai W. [The prevalence of non-alcoholic fatty liver disease and its related risk factors in 1180 school children in Shanghai]. Zhonghua gan zang bing za zhi = Zhonghua ganzangbing zazhi = Chinese journal of hepatology 2007;2007/10/02:644-8.

35. Welsh JA, Karpen S, Vos MB. Increasing prevalence of nonalcoholic fatty liver disease among United States adolescents, 1988-1994 to 2007-2010. The Journal of Pediatrics 2013;162:496-500.

36. Yoo J, Lee S, Kim K, Yoo S, Sung E, Yim J. Relationship between insulin resistance and serum alanine aminotransferase as a surrogate of NAFLD (nonalcoholic fatty liver disease) in obese Korean children. Diabetes Research and Clinical Practice 2008;2008/06/24:321-6.

37. Zhang H, Li Y, Guan X. [Studies on fatty liver, blood glucose and related factors in simple obese children]. Zhonghua yu fang yi xue za zhi [Chinese journal of preventive medicine] 2001;2002/01/05:336-7.

38. Zou CC, Liang L, Hong F, Fu JF, Zhao ZY. Serum adiponectin, resistin levels and non-alcoholic fatty liver disease in obese children. Endocrine journal 2005;2005/11/15:519-24.

39. Arslan N, Buyukgebiz B, Ozturk Y, Cakmakci H. Fatty liver in obese children: prevalence and correlation with anthropometric measurements and hyperlipidemia. Turk J Pediatr 2005;47:23-7.

40. Bedogni G, Gastaldelli A, Manco M et al. Relationship between fatty liver and glucose metabolism: a cross-sectional study in 571 obese children. Nutr Metab Cardiovasc Dis 2012;22:120-6.

41. Bohte AE, Koot BG, van der Baan-Slootweg OH et al. US cannot be used to predict the presence or severity of hepatic steatosis in severely obese adolescents. Radiology 2012;262:327-34.

42. Boza C, Viscido G, Salinas J, Crovari F, Funke R, Perez G. Laparoscopic sleeve gastrectomy in obese adolescents: results in 51 patients. Surg Obes Relat Dis 2012;8:133-7.

43. Cali AM, Zern TL, Taksali SE et al. Intrahepatic fat accumulation and alterations in lipoprotein composition in obese adolescents: a perfect proatherogenic state. Diabetes Care 2007;2007/08/25:3093-8.

44. Chan DF, Li AM, Chu WC et al. Hepatic steatosis in obese Chinese children. International journal of obesity and related metabolic disorders : journal of the International Association for the Study of Obesity 2004;2004/07/28:1257-63.

45. Chiloiro M, Riezzo G, Chiarappa S et al. Relationship among fatty liver, adipose tissue distribution and metabolic profile in moderately obese children: an ultrasonographic study. Current pharmaceutical design 2008;2008/11/11:2693-8.

46. Ciba I, Widhalm K. The association between non-alcoholic fatty liver disease and insulin resistance in 20 obese children and adolescents. 2007;96:109-12.

47. D'Adamo E, Impicciatore M, Capanna R et al. Liver steatosis in obese prepubertal children: a possible role of insulin resistance. Obesity (Silver Spring, Md ) 2008;2008/02/02:677-83.

48. de Piano A, Prado WL, Caranti DA et al. Metabolic and Nutritional Profile of Obese Adolescents With Nonalcoholic Fatty Liver Disease. Journal of Pediatric Gastroenterology and Nutrition 2007;44.

49. de Piano A, Tock L, Carnier J et al. Negative correlation between neuropeptide Y/agouti-related protein concentration and adiponectinemia in nonalcoholic fatty liver disease obese adolescents submitted to a long-term interdisciplinary therapy. Metabolism: clinical and experimental 2010;2009/11/28:613-9.

50. de Silva KS, Wickramasinghe VP, Gooneratne IN. Metabolic consequences of childhood obesity--a preliminary report. The Ceylon medical journal 2006;2007/02/24:105-9.

51. Denzer C, Thiere D, Muche R et al. Gender-specific prevalences of fatty liver in obese children and adolescents: roles of body fat distribution, sex steroids, and insulin resistance. The Journal of clinical endocrinology and metabolism 2009;2009/09/24:3872-81.

52. Duarte MA, Silva GA. Hepatic steatosis in obese children and adolescents. Jornal de pediatria 2011;2011/04/20:150-6.

53. Dubern B, Girardet JP, Tounian P. Insulin resistance and ferritin as major determinants of abnormal serum aminotransferase in severely obese children. International journal of pediatric obesity : IJPO : an official journal of the International Association for the Study of Obesity 2006;1:77-82.

54. el-Karaksy HM, el-Koofy NM, Anwar GM, el-Mougy FM, el-Hennawy A, Fahmy ME. Predictors of non-alcoholic fatty liver disease in obese and overweight Egyptian children: single center study. Saudi journal of gastroenterology : official journal of the Saudi Gastroenterology Association 2011;2011/01/05:40-6.

55. Eminoglu TF, Camurdan OM, Oktar SO, Bideci A, Dalgic B. Factors related to non-alcoholic fatty liver disease in obese children. The Turkish journal of gastroenterology : the official journal of Turkish Society of Gastroenterology 2008;2008/12/27:85-91.

56. Franzese A, Vajro P, Argenziano A et al. Liver involvement in obese children. Ultrasonography and liver enzyme levels at diagnosis and during follow-up in an Italian population. Digestive Diseases and Sciences 1997;1997/07/01:1428-32.

57. Frelut ML, Emery-Fillon N, Guilland JC, Dao HH, de Courcy GP. Alanine amino transferase concentrations are linked to folate intakes and methylenetetrahydrofolate reductase polymorphism in obese adolescent girls. Journal of Pediatric Gastroenterology and Nutrition 2006;2006/08/01:234-9.

58. Fu JF, Liang L, Wang CL, Hong F, Dong GP, Li Y. [Nonalcoholic steatohepatitis in obese children: the prevalence and possible mechanism]. Zhejiang da xue xue bao Yi xue ban = Journal of Zhejiang University Medical sciences 2006;2006/02/14:64-8.

59. Gupta R, Bhangoo A, Matthews NA et al. The prevalence of non-alcoholic fatty liver disease and metabolic syndrome in obese children. Journal of pediatric endocrinology & metabolism : JPEM 2011;2012/02/09:907-11.

60. Guzzaloni G, Grugni G, Minocci A, Moro D, Morabito F. Liver steatosis in juvenile obesity: correlations with lipid profile, hepatic biochemical parameters and glycemic and insulinemic responses to an oral glucose tolerance test. International journal of obesity and related metabolic disorders : journal of the International Association for the Study of Obesity 2000;2000/07/06:772-6.

61. Hacihamdioglu B, Okutan V, Yozgat Y et al. Abdominal obesity is an independent risk factor for increased carotid intima- media thickness in obese children. The Turkish journal of pediatrics 2011;2011/05/04:48-54.

62. Holterman AX, Browne A, Tussing L et al. A prospective trial for laparoscopic adjustable gastric banding in morbidly obese adolescents: an interim report of weight loss, metabolic and quality of life outcomes. Journal of pediatric surgery 2010;2010/01/29:74-8.

63. Iughetti L, Bacchini E, Dodi I et al. [Liver damage and obesity in pediatric age]. La Pediatria medica e chirurgica : Medical and surgical pediatrics 1996;1996/01/01:57-9.

64. Kawasaki T, Hashimoto N, Kikuchi T, Takahashi H, Uchiyama M. The relationship between fatty liver and hyperinsulinemia in obese Japanese children. Journal of Pediatric Gastroenterology and Nutrition 1997;1997/03/01:317-21.

65. Kim JS, LÃª KA, Mahurkar S, Davis JN, Goran MI. Influence of elevated liver fat on circulating adipocytokines and insulin resistance in obese Hispanic adolescents. 2012;7:158-64.

66. Kim G, Giannini C, Pierpont B et al. Longitudinal effects of MRI-measured hepatic steatosis on biomarkers of glucose homeostasis and hepatic apoptosis in obese youth. Diabetes Care 2013;36:130-6.

67. Koot BG, van der Baan-Slootweg OH, Tamminga-Smeulders CL et al. Lifestyle intervention for non-alcoholic fatty liver disease: prospective cohort study of its efficacy and factors related to improvement. Arch Dis Child 2011;96:669-74.

68. Landau Z, Karplus G, Hanukoglu A, Abiri S, Levy A, Serour F. [Laparoscopic sleeve gastrectomy (LSG) in adolescents with morbid obesity]. Harefuah 2011;150:765-8, 816, 815.

69. Lin YC, Chang PF, Yeh SJ, Liu K, Chen HC. Risk factors for liver steatosis in obese children and adolescents. Pediatrics and neonatology 2010;2010/08/03:149-54.

70. Liu LR, Fu JF, Liang L, Huang K. [Relationship between nonalcoholic fatty liver disease and cardiovascular disease in children with obesity]. Zhongguo dang dai er ke za zhi = Chinese journal of contemporary pediatrics 2010;2010/07/20:547-50.

71. Lopez-Capape M, Lopez-Bermejo A, Alonso Blanco M, Lara Orejas E, Corbaton Blasco J, Barrio Castellanos R. [Fatty liver disease, insulin resistance and adiponectin in an obese pediatric population]. Anales de pediatria (Barcelona, Spain : 2003) 2009;2009/10/24:495-501.

72. Louthan MV, Theriot JA, Zimmerman E, Stutts JT, McClain CJ. Decreased prevalence of nonalcoholic fatty liver disease in black obese children. Journal of Pediatric Gastroenterology and Nutrition 2005;2005/10/06:426-9.

73. Maffeis C, Banzato C, Rigotti F et al. Biochemical Parameters and Anthropometry Predict NAFLD in Obese Children. 2011;53:590-3.

74. Ozkol M, Ersoy B, Kasirga E, Taneli F, Bostanci IE, Ozhan B. Metabolic predictors for early identification of fatty liver using doppler and B-mode ultrasonography in overweight and obese adolescents. European journal of pediatrics 2010;2010/05/26:1345-52.

75. Papandreou D, Rousso I, Malindretos P et al. Are saturated fatty acids and insulin resistance associated with fatty liver in obese children? Clinical nutrition (Edinburgh, Scotland) 2008;2008/02/01:233-40.

76. Perito ER, Tsai PM, Hawley S, Lustig RH, Feldstein VA. Targeted hepatic sonography during clinic visits for detection of fatty liver in overweight children: a pilot study. Journal of ultrasound in medicine : official journal of the American Institute of Ultrasound in Medicine 2013;32:637-43.

77. Perseghin G, Bonfanti R, Magni S et al. Insulin resistance and whole body energy homeostasis in obese adolescents with fatty liver disease. American journal of physiology Endocrinology and metabolism 2006;2006/05/11:E697-E703.

78. Pozzato C, Radaelli G, Dall'Asta C et al. MRI in identifying hepatic steatosis in obese children and relation to ultrasonography and metabolic findings. Journal of Pediatric Gastroenterology and Nutrition 2008;2008/10/15:493-9.

79. Pozzato C, Verduci E, Scaglioni S et al. Liver fat change in obese children after a 1-year nutrition-behavior intervention. Journal of Pediatric Gastroenterology and Nutrition 2010;2010/06/22:331-5.

80. Radetti G, Kleon W, Stuefer J, Pittschieler K. Non-alcoholic fatty liver disease in obese children evaluated by magnetic resonance imaging. Acta Paediatr 2006;95:833-7.

81. Reinehr T, Roth CL. Fetuin-A and its relation to metabolic syndrome and fatty liver disease in obese children before and after weight loss. The Journal of clinical endocrinology and metabolism 2008;2008/08/30:4479-85.

82. Reinehr T, Schmidt C, de Sousa G, Andler W. Association between leptin and transaminases: 1-year follow-up study in 180 overweight children. Metabolism: clinical and experimental 2009;2009/03/24:497-503.

83. Reinehr T, Toschke AM. Onset of puberty and cardiovascular risk factors in untreated obese children and adolescents: a 1-year follow-up study. Archives of pediatrics & adolescent medicine 2009;2009/08/05:709-15.

84. Reinehr T, Kleber M, Toschke AM, Woelfle J, Roth CL. Longitudinal association between IGFBP-1 levels and parameters of the metabolic syndrome in obese children before and after weight loss. International journal of pediatric obesity : IJPO : an official journal of the International Association for the Study of Obesity 2011;2011/01/05:236-43.

85. Reinehr T, Woelfle J, Wunsch R, Roth CL. Fibroblast growth factor 21 (FGF-21) and its relation to obesity, metabolic syndrome, and nonalcoholic fatty liver in children: a longitudinal analysis. The Journal of clinical endocrinology and metabolism 2012;97:2143-50.

86. Rocha R, Cotrim HP, Bitencourt AG et al. Nonalcoholic fatty liver disease in asymptomatic Brazilian adolescents. World journal of gastroenterology : WJG 2009;2009/01/20:473-7.

87. Ruiz-Extremera Ãn, Carazo Ãn, SalmerÃ³n Ãn et al. Factors Associated With Hepatic Steatosis in Obese Children and Adolescents. 2011;53:196-201.

88. Sagi R, Reif S, Neuman G, Webb M, Phillip M, Shalitin S. Nonalcoholic fatty liver disease in overweight children and adolescents. Acta paediatrica (Oslo, Norway : 1992) 2007;2007/07/28:1209-13.

89. Sartorio A, Del Col A, Agosti F et al. Predictors of non-alcoholic fatty liver disease in obese children. Eur J Clin Nutr 2006;61:877-83.

90. Shi HB, Fu JF, Liang L et al. [Prevalence of nonalcoholic fatty liver disease and metabolic syndrome in obese children]. Zhonghua er ke za zhi Chinese journal of pediatrics 2009;2009/07/04:114-8.

91. Tazawa Y, Noguchi H, Nishinomiya F, Takada G. Effect of weight changes on serum transaminase activities in obese children. Acta paediatrica Japonica; Overseas edition 1997;1997/04/01:210-4.

92. Tock L, Prado WL, Caranti DA et al. Nonalcoholic fatty liver disease decrease in obese adolescents after multidisciplinary therapy. European journal of gastroenterology & hepatology 2006;2006/11/14:1241-5.

93. Wiegand S, Keller KM, Robl M et al. Obese boys at increased risk for nonalcoholic liver disease: evaluation of 16,390 overweight or obese children and adolescents. Int J Obes (Lond) 2010;34:1468-74.

94. Xanthakos S, Miles L, Bucuvalas J, Daniels S, Garcia V, Inge T. Histologic spectrum of nonalcoholic fatty liver disease in morbidly obese adolescents. Clinical gastroenterology and hepatology : the official clinical practice journal of the American Gastroenterological Association 2006;2006/02/14:226-32.
